# Robotic Cardiac Surgery: The Future Gold Standard or An Unnecessary Extravagance?

**DOI:** 10.21470/1678-9741-2019-0194

**Published:** 2019

**Authors:** Amer Harky, Syed Mohammad Asim Hussain

**Affiliations:** 1 Department of Cardiothoracic Surgery, Liverpool Heart and Chest Hospital, Liverpool, United Kingdom

The shift towards minimally invasive surgery has paved the way for robotic technologies being used to perform operations. Robots have frequently been used for prostatectomy and hysterectomy, whereas their application in cardiac surgery is confined to specialized, high-volume centres^[1]^.

Robotic cardiac surgery utilizes small port incisions avoiding the need for a full sternotomy. This provides benefits in terms of less pain, less bleeding, earlier discharge, quicker recovery, and improved cosmesis. Traditional, video-assisted, or laparoscopic surgeries have many of these advantages, but they have technical limitations due to the use of long-shafted instruments and the fulcrum effect.

Robotic surgery has technical advantages as it improves dexterity by allowing the instruments to move with six degrees of freedom. Other advantages include avoidance of tremor and ambidexterity. Additionally, the three-dimensional (3-D) high-definition imaging greatly improves visualization. The enhanced visual feedback, through observation of tissue displacement and deformation, substitutes for the lack of tactile feedback^[2]^.

In cardiac surgery, robotic technology has most commonly been used to perform mitral valve surgery and coronary artery bypass surgery^[3]^.

## ROBOTIC MITRAL SURGERY

The enhanced 3-D imaging provided by robotic surgery is especially useful in mitral valve surgery as it provides unparalleled visualization of the valve leaflets and the sub-valvular structures.

The first mitral valve repair performed with a robot (the Da Vinci System^®^) occurred in 1998, carried out by Carpentier, and this system gained the Food and Drug Administration approval for mitral valve surgery in 2002^[4]^. Mihaljevic et al.^[5]^ compared the robotic mitral valve repair (n=261) with complete sternotomy (n=114), partial sternotomy (n=170), and mini-thoracotomy approach (n=114). There were no significant differences between the groups with regard to postoperative mortality, pulmonary complications, neurological complications, and renal failure rates. The incidence of atrial fibrillation and pleural effusion was the lowest in the robotic group, which led to a significant reduction in length of hospital stay compared to other groups^[5]^. However, the cardiopulmonary bypass time was significantly longer in the robotic group than in other groups^[5]^. This may be related to the steep learning curve associated with robotic surgery.

In Europe, the rate of use of robotic mitral valve surgery fell for a decade due to the advent of the port access system that allowed mitral valve repair through a mini-thoracotomy. This technique has provided excellent results in a minimally invasive manner; therefore, robotic technology, with its high initial cost and maintenance, had less market share. In contrast, robotic technology for mitral valve repair has gained more traction in the more competitive and privatized healthcare market of the United States of America.

## ROBOTIC CORONARY REVASCULARIZATION SURGERY

Robotic technology can be used in multiple ways for coronary revascularization. It includes totally endoscopic coronary artery bypass (TECAB), whereby the left internal thoracic artery (LITA) is harvested and grafted onto the left anterior descending (LAD) artery using a robot. Alternatively, LITA can be harvested using a robot and then a minimally invasive coronary artery bypass is performed, whereby LITA is hand-sewn to LAD via a mini-thoracotomy. It can be performed on both the beating heart (off-pump) and the arrested heart (on-pump).

The first robotic TECAB was performed in 1998 by Loulmet, using the Da Vinci System^®[^^6]^. Since then, more than 1,000 robotic assisted bypass surgeries have been performed. The results of 326 patients undergoing robotic TECAB showed mortality rate of 0.6%, stroke rate of 2%, perioperative myocardial infarction of 2.5%, and long-term freedom from major adverse cardiac events reported as 81% in the first postoperative 5 years^[7]^. However, 14% of the cases had to be converted to a larger incision, such as sternotomy or mini-thoracotomy^[7]^.

Robotic technology has the potential to provide a new standard of care for coronary revascularization. The new treatment strategy would combine the most beneficial aspects of coronary artery bypass grafting (CABG) (i.e. LITA to LAD anastomosis) and percutaneous coronary intervention (PCI) (stents to other coronaries), resulting in completely minimally invasive hybrid revascularization. There is definitive evidence that LITA to LAD anastomosis significantly improves survival compared to PCI.

PCI with drug-eluting stents is of comparable efficacy to vein grafts and is much less invasive. Theoretically, this strategy maximizes the benefit to the patient and minimizes the risks.

A randomized trial to compare this hybrid strategy with conventional CABG would still be warranted as the type of coronary arteries that receive vein grafts are different from the coronaries that are stented due to patient selection.

## ECONOMIC FEASIBILITY

Robotic surgery has comparable outcomes to conventional surgery with regard to mortality and major adverse events. It has benefits in terms of shorter intensive care and length of hospital stay, lower need for blood transfusion, and decreased pain. These benefits are also provided by minimally invasive mitral and bypass surgeries; therefore, robotic surgery will need to compete for a growth in the market share.

Its widespread use is currently hindered by high initial capital investment and ongoing maintenance costs. The purchase price can exceed US$2 million with a US$100,000 annual service contract^[8]^. It has been criticized for its expense with an average cost per patient up to US$45,914^[8]^. The other major limitation in using robots is the steep learning curve associated with it; a competent operator requires 150-250 procedures to become adept^[8]^.

However, proponents argue that the costs can be compensated by the decreased duration of intensive care and hospital stay, with Morgan et al. suggesting that total operational hospital costs did not significantly increase with robotic technology^[9]^. Advancements in technology have the potential to decrease the learning time required. As many of the patents of the Da Vinci System^®^ have expired, companies are producing 2^nd^ generation robots that may be more versatile, compact, and easier to use^[10]^.

## CONCLUSION

Robotic technology offers an exciting alternative to conventional surgery with potential for a hybrid bypass operation. It also provides a minimally invasive option where conventional endoscopic surgery may technically be too difficult. However, it would need to prove itself as non-inferior to conventional surgery with regard to short- and long-term outcomes in randomized control trials. Then, it would need to further prove itself as an economically viable option.
